# Image-Guided Neutron Capture Therapy Using the Gd-DO3A-BTA Complex as a New Combinatorial Treatment Approach

**DOI:** 10.1155/2018/3727109

**Published:** 2018-11-01

**Authors:** Ki-Hye Jung, Ji-Ae Park, Jung Young Kim, Mi Hyun Kim, Seyoung Oh, Hee-Kyung Kim, Eun-Ji Choi, Han-Jun Kim, Sun Hee Do, Kyo Chul Lee, Kyeong Min Kim, Yong Jin Lee, Yongmin Chang

**Affiliations:** ^1^Division of Applied RI, Korea Institute of Radiological and Medical Science, Seoul 139-706, Republic of Korea; ^2^BK21 Plus KNU Biomedical Convergence Program, School of Medicine, Kyungpook National University, Daegu 700-721, Republic of Korea; ^3^College of Veterinary Medicine, Konkuk University, Seoul 05029, Republic of Korea; ^4^Department of Molecular Medicine, School of Medicine, Kyungpook National University, Daegu 700-721, Republic of Korea; ^5^Department of Radiology, Kyungpook National University Hospital, Daegu 700-721, Republic of Korea

## Abstract

Gadolinium-neutron capture therapy (Gd-NCT) is based on the nuclear capture reaction that occurs when ^157^Gd is irradiated with low energy thermal neutrons to primarily produce gamma photons. Herein, we investigated the effect of neutron capture therapy (NCT) using a small molecular gadolinium complex, Gd-DO3A-benzothiazole (Gd-DO3A-BTA), which could be a good candidate for use as an NCT drug due to its ability to enter the intracellular nuclei of tumor cells. Furthermore, MRI images of Gd-DO3A-BTA showed a clear signal enhancement in the tumor, and the images also played a key role in planning NCT by providing accurate information on the *in vivo* uptake time and duration of Gd-DO3A-BTA. We injected Gd-DO3A-BTA into MDA-MB-231 breast tumor-bearing mice and irradiated the tumors with cyclotron neutrons at the maximum accumulation time (postinjection 6 h); then, we observed the size of the growing tumor for 60 days. Gd-DO3A-BTA showed good therapeutic effects of chemo-Gd-NCT for the *in vivo* tumor models. Simultaneously, the Gd-DO3A-BTA groups ([Gd-DO3A-BTA(+), NCT(+)]) showed a significant reduction in tumor size (*p* < 0.05), and the inhibitory effect on tumor growth was exhibited in the following order: [Gd-DO3A-BTA(+), NCT(+)] > [Gd-DO3A-BTA(+), NCT(−)] > [Gd-DO3A-BTA(−), NCT(+)] > [Gd-DO3A-BTA(−), NCT(−)]. On day 60, the [Gd-DO3A-BTA(+), NCT(+)] and [Gd-DO3A-BTA(−), NCT(−)] groups exhibited an approximately 4.5-fold difference in tumor size. Immunohistochemistry studies demonstrated that new combinational therapy with chemo-Gd-NCT could treat breast cancer by both the inhibition of tumor cell proliferation and induction of apoptosis-related proteins, with *in vivo* tumor monitoring by MRI.

## 1. Introduction

Neutron capture therapy (NCT) is a well-known approach to cancer treatment based on the accumulation of neutron capture agents at the tumor site [1], followed by irradiation with thermal neutrons. NCT is a very effective technique for cancer treatment because the thermal neutrons with low energy do not cause damage to normal cells that lack the neutron capture compounds. This technique thus provides a solution to the major problem of radiation therapy, which is the radiation-induced damage to normal tissue. Therefore, the strategy for the accumulation of NCT compounds specifically within the tumor is critical to avoid damage to normal tissues [2–5].

Gadolinium (^157^Gd)-based NCT (Gd-NCT) has generated recent interest as a cancer treatment due to the following merits. (i) The ^157^Gd atom captures neutrons (^157^Gd + n_th_ (0.025 eV) ⟶ [^158^Gd] ⟶^158^Gd + γ + 7.94 MeV) across a very large cross section (*σ*_th_ = 240,000 b). (ii) The kinetic energy of Gd-NCT, with a mixture of low- and high-energy ionizing particles, is more uniformly distributed throughout tumor tissues, and can be used to solve the shortcomings of heterogeneous *in vivo* dose distribution. (iii) ^157^Gd complexes are widely developed as magnetic resonance imaging (MRI) contrast agents by chelation chemistry and have been routinely used in clinical applications including Gadovist® (gadobutrol), Dotarem® (gadoterate meglumine) and Omniscan® (gadodiamide) [6]. Gadolinium MRI contrast agents could thus be first considered as NCT agents, but it is difficult to specifically target tumor cells *in vivo*. These agents do not accumulate well in tumor tissue during neutron irradiation after an intravenous injection for therapy, as shown in Table 1. The data show high uptake of the contrast agents into tumor cells at 5 mins, but they exhibit very low uptake at 2 hrs. To obtain success with ^157^Gd-NCT, ^157^Gd must be transferred into tumor cells at high concentrations during neutron irradiation. In a previous study, a 50–200 *μ*g ^157^Gd/g tumor was reported as an effective cancer treatment.

For the ^157^Gd neutron capture reaction, the majority of the energy is released as long-range gamma radiation, while, 0.63% of the time, this emission occurs as Auger and conversion electrons. Auger electrons generated from Gd-NCT have strong *in vivo* cytotoxicity by high linear energy transfer (LET), which can induce DNA double-strand breaks (DSB) and restrain the proliferation of tumor cells. Thus, increase in the therapeutic effects is observed when Gd atoms are highly internalized into tumor cells [18, 19].

As therapeutic candidates, various benzothiazoles deserve special attention, as they are known to possess diverse biological properties such as anti-inflammatory, antimicrobial, and anticancer effects. Some of the compounds containing the benzothiazole system are in clinical usage for the treatment of various diseases/disorders [20]. In our previous study, the complexes have not only displayed tumor specificity but also enhanced intracellular MR images of the cytosol and nuclei of a series of tumor cells. The antiproliferative activity of Gd-DO3A-BTA (1), which contains a chelating moiety (DO3A) and a chemoagent region (BTA), was demonstrated by determining the *in vitro* growth inhibition values (GI_50_ and TGI) and monitoring tumor volume regression *in vivo*. In particular, Gd-DO3A-BTA has been reported to specifically accumulate intracellularly in tumors arising from MDA-MB-231 breast cancer cells [9, 21]. On the basis of this result, we make attempts to treat *in vivo* tumor tissue by neutron beam irradiation with a medical cyclotron, preserving the high tumor uptake of ^157^Gd complexes.

## 2. Results

### 2.1. *In Vivo* MR Imaging

**Figure 1:**
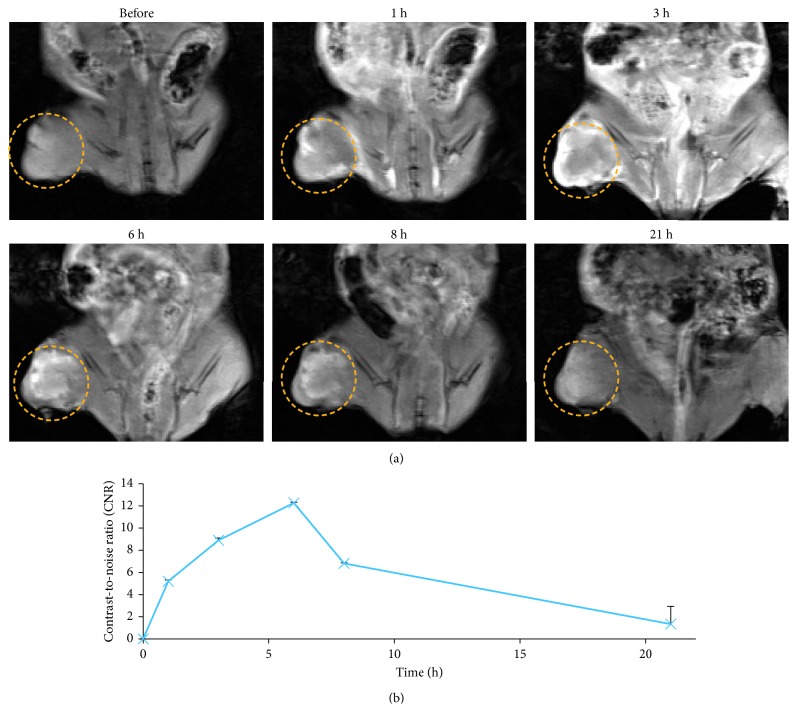
(a) *In vivo* MR images of nude mice with MDA-MB-231 tumors after intravenous injection of Gd-DO3A-BTA. Tumors are indicated with circles. (b) Contrast-to-noise ratio (CNR) as a function of time.

Figure 1(a) shows the *in vivo* T1-weighted MR images of mice that were injected with Gd-DO3A-BTA (0.1 mmol Gd/kg) via the tail vein. T1-weighted MR images were used because the T1 shortening effect is more dominant at the relatively low Gd concentrations used (∼0.1 mmol Gd/kg). The MR image reveals clear tumor enhancement, which increased for 6 h before gradually decreasing (Figure 1(a)). This result is consistent with the biodistribution data reported in our previous study [9], demonstrating that we can effectively define the starting point of NCT with *in vivo* MR imaging.

### 2.2. Gd-NCT Inhibited Tumor Growth with Gd-DO3A-BTA

**Figure 2:**
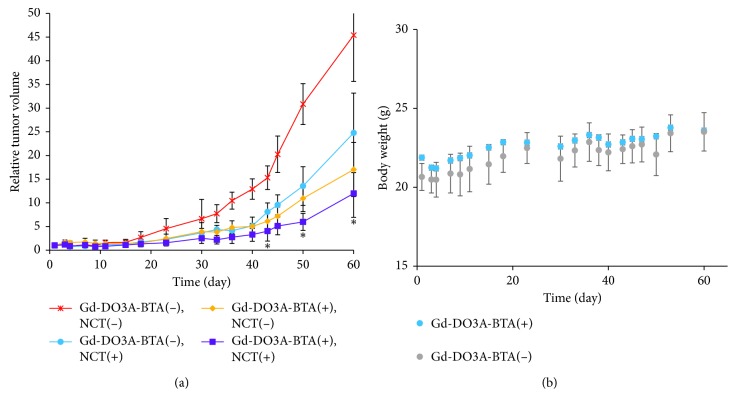
(a) Time-course changes in the relative tumor volume after neutron irradiation. Data are expressed as the mean ± SD (*n*=5), ^*∗*^*p* < 0.05 compared to other groups. (b) Relative changes in the body weight of the mice.

**Figure 3:**
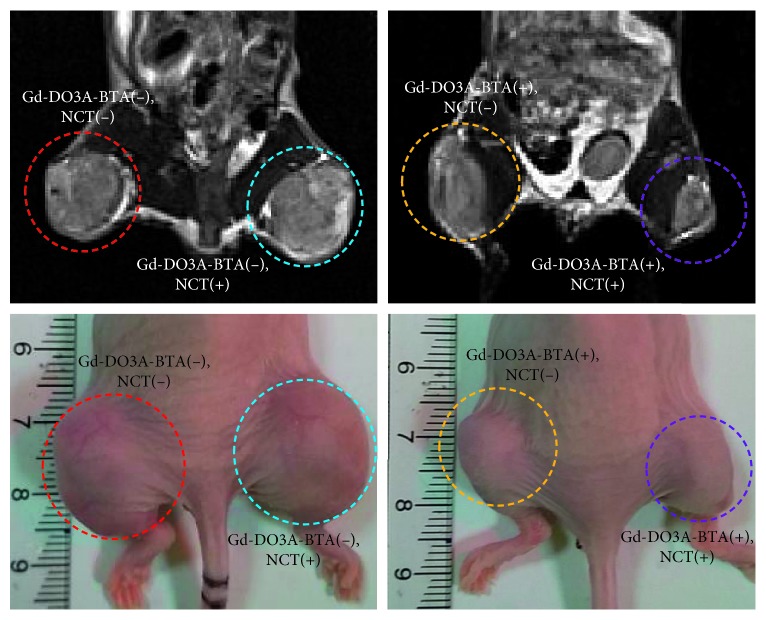
MRI (above) and morphological findings (below) of tumors at 60 days after Gd-NCT. [Gd-DO3A-BTA(+), NCT(+)], Gd-DO3A-BTA administered and neutron irradiated; [Gd-DO3A-BTA(+), NCT(−)], Gd-DO3A-BTA administered and non-neutron irradiated; [Gd-DO3A-BTA(−), NCT(+)], non-Gd administered and neutron irradiated; [Gd-DO3A-BTA(−), NCT(−)], non-Gd administered and non-neutron irradiated.

A small animal study was performed in which mice were irradiated with a neutron beam at 6 h p.i. to determine the optimal time at which the highest uptake of Gd-DO3A-BTA into the tumor tissue occurred. All other tissues, except the tumor tissues, were covered with a plastic box of Teflon to protect them from irregular showering with neutron beams.  Figure 2(a) shows the time-course of change in tumor volume after irradiation with neutron beam for 60 days. The Gd-DO3A-BTA-injected and neutron-irradiated mice group ([Gd-DO3A-BTA(+), NCT(+)]) showed a significant decrease in tumor size than the nontreated groups ([Gd-DO3A-BTA(+), NCT(−)], [Gd-DO3A-BTA(−), NCT(+)], and [Gd-DO3A-BTA(−), NCT(−)]). Four groups began to show significant changes in tumor volume on day 15. In particular, the difference between [Gd-DO3A-BTA(+), NCT(+)] and nontreated groups on days 42, 50, and 60 was significant (*p* < 0.05). The tumor growth suppression was observed in the following order: [Gd-DO3A-BTA(+), NCT(+)] > [Gd-DO3A-BTA(+), NCT(−)] > [Gd-DO3A-BTA(−), NCT(+)] > [Gd-DO3A-BTA(−), NCT(−)]. On day 60, [Gd-DO3A-BTA(+), NCT(+)] and [Gd-DO3A-BTA(−), NCT(−)] groups showed an approximately 4.5-fold difference in tumor size. The mean tumor volume (relative tumor volume) of the [Gd-DO3A-BTA(+), NCT(+)] group was 11.99 ± 5.05, which was much smaller than that in the other groups (*p* < 0.05) on day 60. Due to the tumor suppressive effects of BTA, the antitumor activity of Gd-DO3A-BTA alone (no neutron beam irradiation) was higher than that of neutron irradiation alone. Consistent with earlier reports [10, 22, 23], the neutron irradiation [Gd-DO3A-BTA(−), NCT(+)] alone group also showed some inhibition of tumor growth than the group that did not receive either treatment [Gd-DO3A-BTA(−), NCT(−)]. After irradiation by neutron beams, changes in the body weight of the mice were measured as well as tumor volume. As shown in Figure 2(b), no significant weight loss was observed, suggesting the safety of Gd-DO3A-BTA for NCT. The suppression of tumor growth could be clearly seen from the MR images and morphological findings shown in Figure 3.

### 2.3. Gd-NCT Inhibited Tumor Cell Proliferation and Induced Tumor Cell Apoptosis with Gd-DO3A-BTA

**Figure 4:**
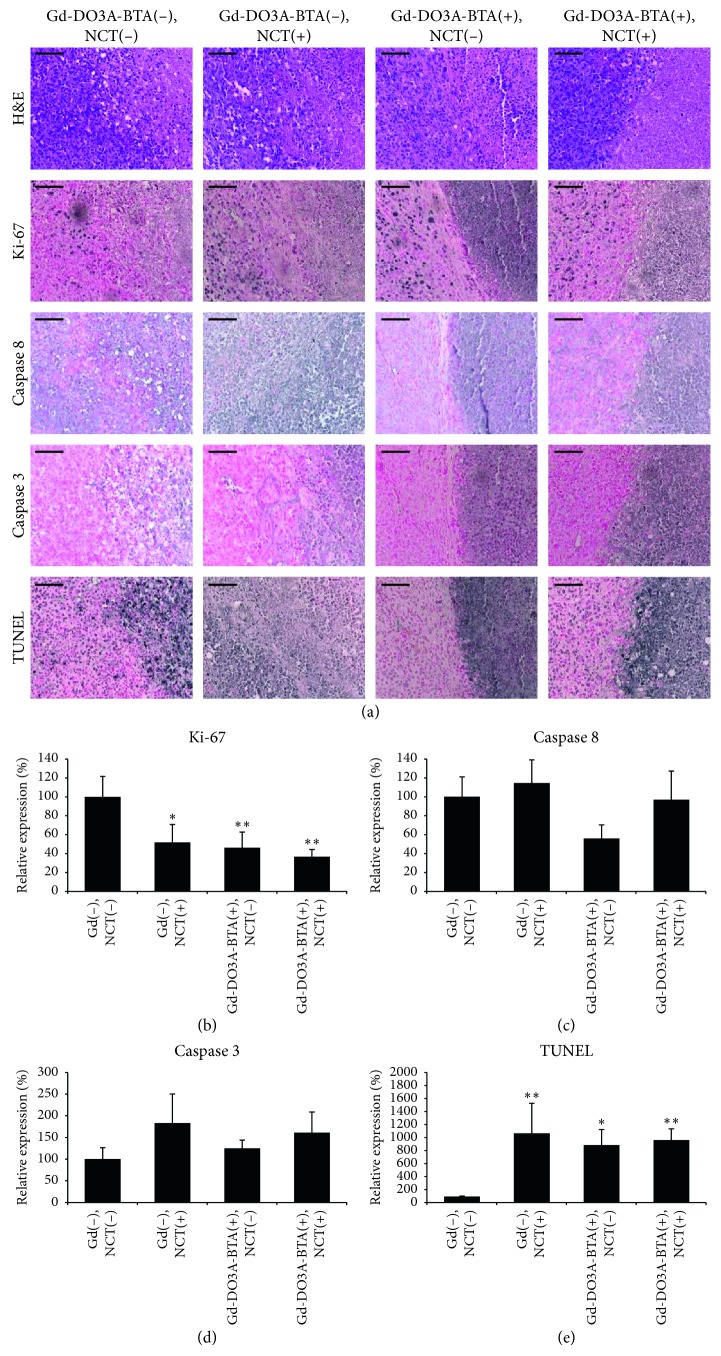
(a) Representative images of hematoxylin and eosin (H&E) staining and immunohistochemical staining of Ki-67, caspase 3, caspase 8, and TUNEL in tumor samples. Graphic plots show an increase in apoptotic cells as determined by (b) Ki-67, (c) caspase 8, (d) caspase 3, and (e) TUNEL staining in Gd-NCT. Scale bars on the images represent 100 *μ*m. ^*∗*^*p* < 0.05; ^*∗∗*^*p* < 0.01 compared to [Gd-DO3A-BTA(−), NCT(−)].

The *in vivo* antitumor activity of Gd-DO3A-BTA in NCT was further verified with histological evaluation and immunohistochemistry, as shown in Figure 4. H&E staining of tumor sections showed proliferating tumor cells and necrotic regions. There were no apparent differences in growth patterns and areas of necrosis between groups. To assess the effect of Gd-NCT on tumor suppression and apoptosis, immunohistochemistry for Ki-67, cleaved caspase 3, and caspase 8, as well as TUNEL staining, was performed. The expression of the proliferation marker Ki-67 was significantly decreased in the [Gd-DO3A-BTA(+), NCT(+)] group compared to that of the [Gd-DO3A-BTA(−), NCT(−)] group. All irradiated groups including [Gd-DO3A-BTA(+), NCT(+)] and [Gd-DO3A-BTA(−), NCT(+)] showed a decrease in Ki-67 expression, indicating that the neutron beam irradiation has tumor suppressive effects. In addition, the [Gd-DO3A-BTA(+), NCT(−)] group also showed decreased Ki-67 expression, implying the antitumor activity of BTA without neutron beam irradiation. Caspase 8 acts as an initiator of apoptosis and is involved in the extrinsic pathway, whereas caspase 3 acts as an effector of apoptosis [24]. The expression of cleaved caspase 3 and 8 did not show significant differences between groups. However, the expressions of both cleaved caspase 3 and 8 were slightly increased in the irradiated groups [Gd-DO3A-BTA(+), NCT(+)] and [Gd-DO3A-BTA(−), NCT(+)] compared to that in the nonirradiated [Gd-DO3A-BTA(+), NCT(−)] and [Gd-DO3A-BTA(−), NCT(−)] groups. Moreover, the evaluation of apoptotic cell death by TUNEL staining revealed that the percentage of cells undergoing apoptosis was greater in all treated groups, including the [Gd-DO3A-BTA(+), NCT(+)] group. These results correlate with the finding that the [Gd-DO3A-BTA(+), NCT(+)] group had the smallest tumor volumes, suggesting that the reduced Ki-67 activity and increased apoptotic cell death after BTA injection and neutron beam irradiation induced tumor growth suppression.

## 3. Discussion

For the success of Gd-NCT as a therapeutic modality for cancer, it is important to effectively deliver and sufficiently accumulate Gd into tumors. For an effective *in vivo* Gd-NCT, an optimal dose of 50–200 *µ*g·Gd/g tumor tissue has been reported [11, 25]. As shown in Table 1, 0.1 mmol/kg of Gd-DO3A-BTA administered as an intravenous injection resulted in approximately 221 *µ*g of Gd/g tumor tissue [9]. This uptake was approximately 1.4 times higher than that reported previously (158 *µ*g·Gd/g tumor tissue) with an intravenous injection of Gd-DTPA-encapsulated liposomes [11, 12].

Various nano-based formulations have been developed to deliver sufficient amounts of Gd into tumor cells for Gd-NCT, and these include nanoparticles [15–17, 26–28], liposomes [10, 11, 12], emulsions [13, 29], and microcapsules [7, 30]. Most of these particles were designed to be approximately 100 nm in size to avoid high uptake by the reticuloendothelial system (RES). However, these particles have limited specific tumor targeting ability, since their delivery completely depends on the enhanced permeability and retention (EPR) effect around tumor sites [11, 14, 31]. Furthermore, these nanoparticles often show high *in vivo* instability by releasing Gd from the nano-based formulations due to the high solubility of Gd-DTPA in an aqueous solution [11, 13, 15]. Therefore, some nano-based formulations for Gd-NCT use a lipophilic complex such as Gd-DPTA/pLL or Gd-acetylacetonate (Gd-acac) to prevent the possible release of Gd [11, 29]. However, as shown in Table 1, these nano-based formulations can be injected only by intraperitoneal or intratumoral methods and cannot be administered intravenously.

Gd-DO3A-BTA, however, is readily administered intravenously. Furthermore, Gd-DO3A-BTA has a relatively small molecular weight (M.W. 767) and high chemical stability, which makes it a better tumor-theranostic agent for MR image-guided NCT with a good therapeutic dosage for clinical use. However, for the success of Gd-NCT, Gd must be transferred into tumors at high concentrations during neutron irradiation. The MR imaging data demonstrated that Gd-DO3A-BTA preferentially accumulated in tumor tissue *in vivo*. Gd-DO3A-BTA delivers more Gd to tumor tissue than conventional Gd-based MRI contrast agents, which may result in a more efficient uptake by the tumor. The effective tumor ablation by Gd-DO3A-BTA could therefore be associated with the high accumulation of Gd in tumor tissue, which in turn captures sufficient thermal neutrons to kill the tumor. Although enriched ^157^Gd compound would be preferable for Gd-NCT, in this experiment, we utilized the natural Gd compound. The natural abundance of ^157^Gd is only 15.65%, while enriched compounds may contain up to 90% ^157^Gd [20]. Therefore, in the near future, it would be important to perform Gd-NCT experiment with enriched ^157^Gd compounds to confirm the current results using the natural Gd compound. The tumors in mice that were irradiated and injected with Gd-DO3A-BTA were much smaller than those observed in other groups, demonstrating that the Gd-DO3A-BTA could significantly enhance the therapeutic effect of Gd-NCT. In addition, the tumor-therapeutic effects of Gd-DO3A-BTA in combination with NCT were confirmed by MR imaging and histological evaluation. Furthermore, the tumor suppressive effects were evident even after 60 days, suggesting that early treatment of the tumor with a Gd-DO3A-BTA injection, and NCT has the potential to suppress further tumor progression. However, the limitation of the current study should be mentioned. In this study, the neutron beam was generated by irradiating a proton beam upon a beryllium target using an MC-50 cyclotron, and the thermal neutron flux was low. Because the high flux of thermal neutrons is usually used for NCT, it is necessary to perform NCT experiment with low energy neutron at high flux to be sure that the effect shown in the current study is due to NCT.

## 4. Conclusions

The present study presented the potential for Gd-NCT using low molecular weight Gd chelate as a new combinatorial chemo-NCT approach in cancer treatment. Despite the simple chemical structure of the metal complex, Gd-DO3A-BTA shows a high therapeutic effect when used in Gd-NCT against solid tumors. The high accumulation of Gd-DO3A-BTA in tumor tissues effectively damaged the tumor cells. Furthermore, as an MR contrast agent, Gd-DO3A-BTA can guide NCT and monitor tumor growth via MR imaging.

## 5. Materials and Methods

### 5.1. General

The Gd-DO3A-BTA (nonenriched Gd) was prepared as described previously [10, 21]. All animal experiments were conducted in compliance with the Guidelines for the Care and Use of Research Animals under protocols approved by the Korea Institute of Radiological and Medical Sciences (KIRAMS) Animal Studies Committee. The neutron beam irradiations including low content thermal neutrons were performed using an MC-50 cyclotron (Scanditronix, Sweden, 1985). When the neutron beam was generated by irradiating a proton beam (20 *μ*A, 35 MeV) upon a beryllium target of 15 mm thickness, the following results were previously reported: the thermal neutron (0.24% of total neutrons) flux was approximately 1.94 × 10^4^ n/cm^2^·sec and the cross section was 13.79 ± 0.45 barn (a). At that time, the absorbed dose of neutron beams was 9.36–8.69 cGy/min (including the gamma ray of 1.42–1.57 cGy/min) at the depth of 15–30 mm with the field size of 26 × 26 cm^2^ (b). The irradiation time of neutron beams in our studies was typically performed as the standard point of approximately 1 Gy/12 min [32, 33].

### 5.2. Tumor Model

The human breast adenocarcinoma cancer cell line MDA-MB-231 (ATCC CRM-HTB-26) was purchased from the American Type Culture Collection (ATCC). MDA-MB-231 is a highly aggressive, invasive, and poorly differentiated triple-negative breast cancer (TNBC; estrogen receptor (ER), progesterone receptor (PR), and HER2 (human epidermal growth factor receptor 2)) cell line. The cells were maintained in RPMI-1640 containing 10% fetal bovine serum (FBS) and 1% antibiotics and were grown in a humidified incubator at 37°C and 5% CO_2_. MDA-MB-231 tumor cells (1×10^8^ cells·mL^−1^) suspended in RPMI-1640 medium without FBS, and antibiotics were injected into the subcutaneous tissue (sc) of female BALB/c nude mice (aged 6 weeks, 18–25 g of body weight) in both legs. One week after tumor cell implantation, the mice were divided into two groups (*n*=5/group). To compare the effect of chemotherapy and NCT on solid tumor, the mice were administered (A) none or (B) Gd-DO3A-BTA (0.1 mmol/kg) intravenously through the tail vein. Only the right-sided tumors were irradiated by neutrons.

### 5.3. *In Vivo* MR Imaging

The mice were anesthetized with 1.5% isoflurane in oxygen. Tumor measurements were made before and after injection of 0.1 mmol·Gd/kg via the tail vein. MR images were taken with a 3 T MR unit (Magnetom Tim Trio, Siemens Medical solution, Erlangen, Germany) using an animal coil. The T1-weighted fast spin-echo imaging was performed under the following conditions: repetition time = 9.9 ms; echo time = 3.2 ms; 10 mm field of view; 256 × 256 matrix size; 1 mm slice thickness; and average = 3.

The contrast to noise ratio (CNR) was defined as the difference in signal-to-noise ratio (SNR) between adjacent anatomic structures.

(1) $$\begin{array}{c} \text{CNR}={\text{SNR}}_{\text{post}}-{\text{SNR}}_{\text{pre}}. \end{array}$$

### 5.4. *In Vivo* Gd-NCT

**Figure 5:**
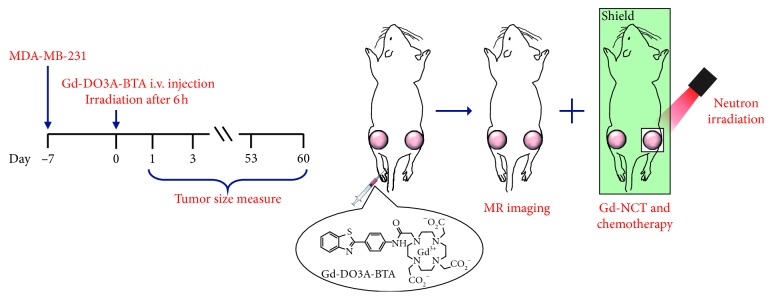
Schematic illustration of *in vivo* Gd-NCT. Except for the right tumor region irradiated by neutrons, the whole body was shielded by Teflon.

Gd-DO3A-BTA was administered intravenously as a bolus (0.1 mmol/kg) into the tail vein of female mice with MDA-MB-231 tumors. After 6 h, the mice were locally irradiated on one side with a 0.3 Gy neutron beam, while the other side was shielded using Teflon as shown.  Figure 5 shows the overall experimental scheme for Gd-NCT. The tumor size was measured before and after irradiation, and the volume (*V*) was calculated using the following equation:

(2) $$\begin{array}{c} V=\frac{a\times b^{2}}{2}, \end{array}$$

where *a* and *b* are the major and minor axes of the tumor measured by a caliper.

### 5.5. Tissue Preparation and Histological Evaluation

The animals were sacrificed after 60 days, and tumor tissues were obtained. The samples were fixed in 10% neutral buffered formalin, processed, and embedded in paraffin, according to standard procedures. The sections were then stained with hematoxylin and eosin (H&E) using an automated processor (Tissue-Tek, PrismaE2, Sakura, Japan). The images were acquired using a digital scanner (Panoramic MIDI, 3D HISTECH Ltd, Hungary).

### 5.6. Immunohistochemical Analysis

The tumor samples were serially sectioned (4 *μ*m sections), deparaffinized, and rehydrated. The sections were incubated in 0.3% hydrogen peroxide to quench the endogenous peroxidase after antigen retrieval by heating in 10 mM citrate buffer (pH 6.0). The immunohistochemistry was performed according to the avidin-biotinylated-HRP complex (ABC) method using the Vectastain Elite ABC kit (Vector Laboratories, Burlingame, CA, USA). The primary antibodies used were Ki-67 and caspase 8 (both 1 : 100, Abcam, Cambridge, MA, USA) and caspase 3 (1 : 100, Santa Cruz, Santa Cruz, CA, USA). Apoptosis was assessed by terminal deoxynucleotidyl transferase-mediated dUTP nick-end labeling (TUNEL; Roche Diagnostics, Indianapolis, IN, USA) according to the manufacturer's instructions. All immunohistochemistry reactions and TUNEL staining were visualized by Vector SG (Vector Laboratories) and counterstained with fast nuclear solutions (Vector Laboratories). Staining was quantified using ImageJ image analysis software (NIH, Bethesda, MD, USA).

### 5.7. Statistical Analysis

Data in the manuscript are expressed as the mean and standard deviation (SD), and the significance of the results was analyzed by Student's *t*-test. *p* < 0.05 was considered statistically significant.

## Figures and Tables

**Scheme 1:**
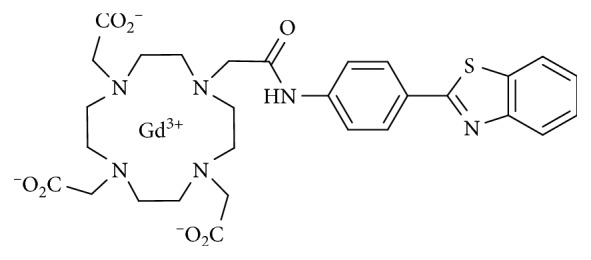
Structure of Gd-DO3A-BTA.

**Table 1:** Various types of gadolinium agents for NCT.

	Form	Injection route	Injection amount (Gd)	Tumor accumulation Gd/g tumor	Highest uptake time (ICP time)	Reference
Low molecules	Gd-DTPA	i.t.	1200 *µ*g/mouse	451 *µ*g/g	5 min	[7]
				5.3 *µ*g/g	24 h	
	Gd-DTPA	i.v.	0.1 mmol/kg	70.39 ± 8.75 *µ*g/g	5 min	[8]
				30.22 ± 4.91 *µ*g/g	2 h	
	Gd-BOPTA	i.v.	0.1 mmol/kg	100.33 ± 7.91 *µ*g/g	5 min	[8]
				40.93 ± 1.83 *µ*g/g	2 h	
	Gd-DO3A-BTA	i.v.	0.1 mmol/kg	221 *µ*g/g	6 h	[9]

Nanoparticles	Liposome	i.v.	N/D	40.277 ± 2.512 *µ*g/g	2 h	[10]
	Liposome	i.v.	20 mg/kg	158.8 ± 115.6 *µ*g/g	12 h	[11, 12]
	Lipid emulsions	i.p.	6 mg/hamster	107 *µ*g/g	48 h	[13]
	Micelles	i.v.	0.02 mmol/kg	3.9% ID/g^a^	10 h	[14]
	Lipid NPs	i.v.	6 mg/hamster	100.7 *µ*g/g	12 h	[15]
	Chitosan NPs	i.t.	2.4 mg/mouse^b^	1766 ± 96 *µ*g/tumor tissue^c^	8 h	[16, 17]
	Chitosan NPs	i.t.	1200 *µ*g/mouse	897.1 *µ*g/g	24 h	[7]

N/D: not determined; i.t.: intratumoral; i.v.: intravenous; i.p.: intraperitoneal. ^a^%ID/g: percentages of the total injected dose per organ weight. ^b^Administered twice by i.t. injection 24 h and 8 h before the assay. ^c^The Gd content in melanoma tissue in mice.

## Data Availability

The data used to support the findings of this study are included within the article.
